# Sexual behaviour, sexually transmitted infections and attitudes to chlamydia testing among a unique national sample of young Australians: baseline data from a randomised controlled trial

**DOI:** 10.1186/1471-2458-14-12

**Published:** 2014-01-08

**Authors:** Melissa Kang, Arlie Rochford, S Rachel Skinner, Adrian Mindel, Marianne Webb, Jenny Peat, Tim Usherwood

**Affiliations:** 1 Discipline of General Practice, University of Sydney, PO Box 154, Westmead, New South Wales 2145, Australia; 2 Discipline of Paediatrics and Child Health, University of Sydney, The Children’s Hospital at Westmead, Locked Bag 4001, Westmead, New South Wales 2145, Australia; 3 Sexually Transmitted Infections Research Centre, (now Western Sydney Sexual Health Centre), University of Sydney, Jefferson House, ParramattaNew South Wales 2150, Australia; 4 Inspire Foundation, 97 Church Street, Camperdown, New South Wales 2050, Australia; 5 Research Consultant, Sydney, PO Box 2192, Tomerong, New South Wales 2540, Australia

**Keywords:** Adolescents, Young people, Chlamydia, Sexually transmitted infections, Health risk behaviour

## Abstract

**Background:**

Chlamydia infection is the most common notifiable sexually transmitted infection (STI) in Australia and mostly affects young people (15 – 25 years). This paper presents baseline data from a randomised controlled trial that aimed to increase chlamydia testing among sexually active young people. The objectives were to identify associations between sexual behaviour, substance use and STI history and explore attitudes to chlamydia testing.

**Methods:**

This study was conducted in cyberspace. Study recruitment, allocation, delivery of interventions and baseline and follow up data collection all took place online. Participants were 16 – 25 years old and resided in Australia. Substance use correlates of sexual activity; predictors of history of STIs; barriers to and facilitators of chlamydia testing were analysed.

**Results:**

Of 856 participants (79.1% female), 704 had experienced penetrative intercourse. Sexually active participants were more likely to smoke regularly or daily, to drink alcohol, or to have binge drunk or used marijuana or other illicit substances recently. Risk factors for having a history of any STI were 3 or more sexual partners ever, 6 or more partners in the past 12 months, condom non-use and being 20 years or older. Almost all sexually active participants said that they would have a chlamydia test if their doctor recommended it.

**Conclusions:**

Sexually active young people are at risk of STIs and may engage in substance use risk behaviours. Where one health risk behaviour is identified, it is important to seek information about others. Chlamydia testing can be facilitated by doctors and nurses recommending it. Primary care providers have a useful role in chlamydia control.

**Trial Registration:**

Australian and New Zealand Trials Registry ACTRN12607000582459

## Background

Most people experience their first partnered sexual interactions in the second decade of life [1]. The average age of first penetrative vaginal intercourse in Australia has been 16 years for females and males for over three decades [2,3]. Despite the initiation of sexual intercourse in the teenage years being normative, the 1980s’ HIV pandemic and changing patterns of sexual relationships among young people have contributed to worldwide concern about adolescent sexual behaviour as a public health issue. Genital chlamydia infection is more prevalent than HIV globally and affects people under 25 years more than any other age group [4]. To address the rapid rise of chlamydia notifications in Australia since 1999, the first (2005 – 2008) and second (2010 – 2013) national sexually transmissible infections (STI) strategies included chlamydia control and young people as specific priorities [5,6]. One of the key components of both strategies was to increase chlamydia testing among target groups.

Research into young people’s sex lives in the public health context has focused on sexual risk behaviour. This has been variously defined as penetrative intercourse without consistent condom use, intercourse with casual partners, having three or more sexual partners in twelve months and/or initiation of sexual intercourse before 16 years of age [7-9]. Many studies have found that adolescent ‘risk behaviours’ tend to cluster, for example smoking, binge drinking and sexual risk behaviours [9,10]. The technical ease with which chlamydia infection can be tested in urine samples has added a new dimension to recent studies, with attention to young people’s interaction with the health system and the barriers and facilitators to testing of prime importance.

This study was one of fifteen projects funded by the first national STI strategy [5] to evaluate chlamydia testing interventions. The aims of this study were to:

– Provide demographic, substance use and sexual history characteristics of a unique sample of young Australians (16 – 25 years).

– Identify associations between sexual behavior, substance use and STI history.

– Report on chlamydia knowledge and attitudes to chlamydia testing.

## Methods

This paper reports on baseline data from a randomised controlled trial (RCT) [11] of 16 – 25 year-old Australians. The study was conducted in cyberspace. A website about chlamydia was launched in March 2007 and invited eligible visitors to participate in a study to promote chlamydia testing. This website was developed with input from 20 youth consultants (16–25 years) who were recruited through professional and collegiate networks. The website was promoted through paid advertising, existing youth-related websites, social networking sites and opportunistic radio interviews. Website traffic was monitored using Google Analytics.

The website invited eligible visitors to participate in the study via clickable links on the homepage and all other webpages. To be eligible, visitors had to be aged 16 – 25 years, reside in Australia, answer ‘yes’ to whether they had ever had penetrative (vaginal or anal) intercourse and provide an email address. Potential participants were taken to a Participant Information Statement, entered a current email address and ticked a consent box. This step took them to the baseline questionnaire housed within the website.

The sampling frame was all eligible visitors to the website, but participation rates were not measurable since the denominator was unknown. The target sample size for the RCT was 1000. A detailed description of the RCT methods has been published elsewhere [11].

One hundred and fifty-two young people who completed baseline data had not had penetrative sex and were not enrolled in the RCT. Their substantial number provided a comparison group when analysing sociodemographic correlates of health risk behaviours, as many differences achieved statistical significance.

The baseline questionnaire collected sociodemographic data, sexual and substance use history, knowledge about chlamydia, attitudes towards chlamydia testing and testing preferences. Some attitude questions explored known barriers to accessing health care for Australian young people [12], such as concerns about confidentiality and owning one’s own ‘Medicare card’. Australia has a universal health insurance scheme (‘Medicare’) and doctors and pathology providers can choose to bill a service directly to Medicare, meaning the patient has no upfront fee. Possessing one’s own card (separate from a family card) is not essential, but can facilitate this direct billing process.

Data were analysed using SPSS v19.0 (IBM, USA). Categorical variables were compared between groups using chi-square tests with Yate’s correction for 2 × 2 tables. Continuous variables were compared using independent samples t-tests. Logistic regression analyses were conducted on the whole sample to assess substance use behaviours that were associated with being sexually active while controlling for age and sex, and on the sexually active group to predict risk factors for STIs.

Ethical approval was obtained from The University of Sydney Human Research Ethics Committee and the trial was registered with the Australian and New Zealand Trials Registry (ACTRN12607000582459).

## Results

Recruitment took place between March 2007 and January 2008 and 856 people aged 16 – 25 years provided complete datasets. Seven hundred and four young people reported they had ever had sexual intercourse and 152 reported they had never had sexual intercourse.

Of the 856 young people, 677 (79.1%) were female, 175 (20.4%) were male and 4 (0.5%) did not state their sex. Mean age was 19.8 years (SD = 3.0). Twenty-seven (3.2%) were either Aboriginal or Torres Strait Islander or both, compared to 3.4% of the general population aged 15 – 24 years [13]. The majority were born in Australia, spoke English at home and were studying and living at home with family. All states and territories of Australia were represented, as were all Australian Standard Geographical Classification groups (major cities, inner regional, outer regional, remote, very remote). The majority (83.3%) were fully engaged in employment and/or education (working or studying *full time* or working *and* studying). Those not fully engaged were working part time or casually (8.3%), looking for work (5.0%), engaged in parenting or domestic duties (1.8%) or did not respond (1.8%). Sixty-three point nine percent had attained a Year 12 certificate (the highest school-based qualification in Australia) or above (27.7% were still in school and could not yet have attained this). Table 1 compares these characteristics between our sample and the general population aged 15 – 24 in Australia.

**Table 1:** **Sociodemographic characteristics of the sample (n = 856) compared to the population aged 15–24 years from the 2006 Census **[15]

	**Sample at baseline 2007**	**Australian population**
	**16 – 25 years**	**15 – 24 years in 2006**
	**Valid percent**	**Percent**
**Sex = female**	79.1	48.7
**Born in Australia**	88.4	84.1
*Born overseas in English speaking country*	*6.7*	*5.3*
*Born overseas in non-English speaking country*	*5.1*	*10.1*
**Aboriginal and/or Torres Strait Islander**	3.2	3.4
**Speak English at home**	95.3	84.0
**State of residence**		
New South Wales	33.0	32.2
Victoria	25.7	24.9
Queensland	17.8	19.9
WA	9.2	10.2
SA	5.7	7.4
Tas	4.0	2.3
ACT	3.4	1.9
NT	1.2	1.1
**Geographic location**		
Major cities	71.5	68.4
Inner Regional	18.5	19.6
Outer Regional	9.1	9.1
Remote	0.7	1.4
Very remote	0.1	0.9
**Occupation**		
Fully engaged in education and/or employment	83.3	83.6*
**Education Level (highest attained)**		
Completed Year 12 or above	63.9	59.1*
**Living situation**		
*At home with parents/guardian*	57.8	
*Away from home (private rental, campus based, with friends)*	41.0	
*Refuge or supported accommodation*	0.7	
*Not stated*	0.5	

*Taken from 2007 Education and Work Australia survey [16].

### Sexually active and non-sexually active respondents compared

The mean age of young people reporting that they had ever had sex at baseline (“sexually active, SA”, n = 704) was significantly higher than the mean age of those reporting never having had sex (‘NSA’ group, n = 152). There were higher proportions of males and of people who spoke English at home in the SA group compared to the NSA group. Logistic regression was performed to explore associations between being sexually active and substance use. Odds ratios were adjusted for age and gender. SA young people were significantly more likely than NSA young people to smoke regularly or daily, to drink alcohol at all or to drink once a week or more, to have binge drunk (5 or more drinks in a row) in the past 2 weeks, and to have used marijuana or other illicit substances in the past 30 days (Table 2).

**Table 2:** Substance use factors associated with being sexually active (n = 856)

	**Sexually active (n = 704)**	**Not sexually active (n = 152)**	Odds ratio (95% CI)*	**P value**
Smoking regularly	29.0%	2.7%	15.1 (5.5, 41.6)	<0.0001
Drinking alcohol ever	93.8%	63.8%	6.6 (4.1, 10.7)	<0.0001
Drinking > once/week	49.0%	12.5%	4.9 (2.9, 8.3)	<0.0001
Binge drinking	58.4%	17.1%	6.2 (3.9, 9.9)	<0.0001
Marijuana in last 30 days	19.2%	5.3%	5.0 (2.3, 10.6)	<0.0001
Illicit drugs last 30 days	12.9%	1.3%	11.1 (2.6, 46.0)	0.001

*Adjusted for age and gender.

Chlamydia knowledge was measured using seven questions, some of which were taken directly, and others adapted from, the National Secondary Students and Sexual Health Survey [14]. SA young people scored significantly higher on composite scores, and for six out of seven questions (Table 3).

**Table 3:** Differences in chlamydia knowledge between sexually active and non-sexually active young people

**Question**	**Sexually active% who answered correctly (n = 704)**	**Not sexually active% who answered correctly (n = 152)**	**p value**
Chlamydia is a sexually transmissible infection that affects only women	89.4	84.7	0.05
Chlamydia can lead to sterility among women	84.7	80.5	0.11
A woman can have chlamydia without any obvious symptoms	91.3	86.0	0.03
A man can have chlamydia without any obvious symptoms	83.4	76.5	0.02
Chlamydia is curable	84.6	77.3	0.03
Chlamydia can be prevented by using condoms when you have sex	91.2	82.0	0.0003
Chlamydia can be tested for with a urine sample	77.1	70.0	0.05
Mean composite score/7	5.3	4.9	0.001

### Characteristics of the sexually active group

The SA group (n = 704) was analysed to explore sexual history (number of sexual partners, condom use, history of chlamydia or other STIs) as well as attitudes to and preferences for chlamydia testing.

The mean age of the SA group was 20.3 years (SD 2.9) and 78.0% were female. Although mean age of males was higher than females, mean age of first penetrative intercourse for females was significantly lower. Compared to females, males had greater numbers of sexual partners ever and in the past 12 months. Among the STIs, gender was significant only for gonorrhoea, where males were more likely to have had a diagnosis than females. There was no difference between males and females with respect to condom use. Table 4 shows sexual history variables by sex.

**Table 4:** Differences in sexual history between females and males

**Variable**	**Females**	**Males**	**p value**
	**N = 547**	**N = 154**	
Mean age (SD)	20.0 years (3.0)	21.4 years (2.6)	p <0.0001
Mean age of first intercourse (SD)	16.1 years (2.3)	16.7 years (2.3)	0.004
Mean no. of years since first intercourse (SD)	3.9 years (3.0)	4.7 years (3.0)	0.002
Number of sexual partners ever			
*One*	22.9%	14.4%	
*Two*	12.3%	11.8%	0.03
*Three to five*	25.8%	20.9%	
*Six to ten*	17.8%	22.9%	
*Eleven or more*	21.2%	30.0%	
Number of sexual partners in last 12 months			
*One*	49.9%	40.4%	
*Two*	19.3%	17.2%	0.02
*Three to five*	20.6%	23.8%	
*Six to ten*	5.4%	12.6%	
*Eleven or more*	4.8%	6.0%	
Use condoms always or mostly	51.6%	56.5%	0.32
History of chlamydia	17.6%	13.9%	0.34
History of HPV	8.1%	9.2%	0.81
History of gonorrhoea	1.3%	5.2%	0.009
History of genital herpes	3.7%	4.5%	0.79
History of HIV	1.1%	0.6%	0.35

One hundred and eighteen young people (16.9%) reported a history of ever being diagnosed with chlamydia at baseline. The next most frequently reported STI was human papillomavirus (defined as ‘*human papillomavirus also called HPV or the wart virus*’) (8.3%) followed by genital herpes (3.9%), gonorrhoea (2.1%) and HIV (1.0%).

One hundred and eighty-eight young people (26.7%) reported having had a chlamydia test in the past six months, of these 70 (37.2%) reported that the test was positive. All but one who reported a positive chlamydia test stated that they had received antibiotic treatment and 65/70 had informed their partner of the result. Forty-seven (67.1%) of those who had tested positive reported that they had returned for a second test. Most who had not returned for a second test stated that insufficient time (to test for re-infection) had passed.

A logistic regression analysis was conducted to predict risk factors for ever having had an STI. Four of the independent variables (age, number of sexual partners in the past 12 months and ever, and condom use) made a statistically significant contribution to the model. Gender, being Aboriginal and/or Torres Strait Islander, speaking English at home and having first intercourse before the age of 16 years were neither significant univariate predictors nor significant when included in the model. The strongest predictor was the number of sexual partners ever. Table 5 shows the adjusted odds ratios for the risk factors for STIs.

**Table 5:** Predictors of STI history in participants who had sexual intercourse ever

**Risk factor**	**Percent (%) in exposed group**	**Percent (%) in unexposed group**	**Unadjusted odds ratio**	**Adjusted odds ratio (95% CI)**	**P value**
**N**	164	524			
Gender (female)*	23.9%	23.5%	0.98	-	
Aboriginal and/or Torres Strait Islander*	38.1%	23.4%	2.41	-	**-**
English at home*	24.2%	11.5%	2.44	-	**-**
**Age 20 years and older**	29.0%	13.1%	2.72	1.95 (1.22, 3.12)	0.005
Age first intercourse < = 15 years*	26.4%	22.6%	1.23	-	
**Condom use never or sometimes**	28.5%	19.7%	1.61	1.48 (1.02, 2.16)	0.04
**Three or more sexual partners ever**	32.3%	7.0%	6.34	4.78 (2.67, 8.54)	<0.0001
**Six or more sexual partners last 12 months**	45.9%	21.1%	2.01	2.25 (1.34, 3.79)	0.002

*Not significant predictors in multivariate logistic regression.

Early initiation of sexual intercourse (< 16 years) was associated with being Aboriginal and/ or Torres Strait Islander (71.4% cf 31.6%, p <0.0001); no/low condom use (54.7% cf 42.7%; p = 0.004); 6 or more partners in the past 12 months (17.5% cf 7.7%; p < 0.0001); smoking regularly (47.9% cf 27.2%, p < 0.0001) and using cannabis (46.0% cf 29.9%; p = 0.001) or illicit drugs (44.6% cf 31.3%, p = 0.02). There was no association between early sex and gender, alcohol use or history of chlamydia testing.

Attitudinal questions explored barriers and facilitators to testing. The strongest facilitators of testing in the SA group were ‘if my doctor recommended it’ (97.4% agreed), ‘because I don’t want to give it to my partner if I have it’ (95.4% agreed), ‘to prevent long term health problems’ (94.5% agreed) and ‘if my partner wanted me to’ (88.5% agreed). Seventy-six percent agreed that they ‘would feel comfortable visiting a doctor or nurse for a chlamydia test’ , although 43.2% also agreed that they ‘would feel too embarrassed to talk to a doctor or a nurse about chlamydia’. Less than half the sample (46.2%) agreed that they did not want to talk about their sexual history with a doctor or nurse. Barriers to testing included being scared of what the test might show (40.0% agreed), their partner/s finding out (25.1% agreed) concerns about cost (36.5% agreed) confidentiality (19.8% agreed), transport (17.3% agreed) and not having their own Medicare card (11.2% agreed). Thirty-three percent agreed that they did not know how to get a chlamydia test. Seventy-nine percent agreed that they would like to see a doctor or nurse the same sex as themselves to get a chlamydia test. Young women were significantly more likely than young men to prefer seeing a doctor of the same sex (83.2% cf 66.0%, p <0.0001) but there were no gender differences in relation to attitudes to testing.

Two hundred and ninety-six young people in the SA group (42.0%) indicated that they would prefer to have a chlamydia test done by their usual general practitioner (GP), and 144 (20.5%) preferred a different GP. One hundred and fifty-five young people (22.0%) preferred to be tested at a sexual health clinic. The remainder preferred a Youth Health Service (4.5%), Family Planning Clinic (2.8%), or did not state a preference (8.2%).

## Discussion

This unique sample, recruited entirely in cyberspace, adds to knowledge about sexual behaviours of young Australians and their socio-demographic and substance use correlates. For those who had had sexual intercourse, the mean age of sexual debut was 16.2 years. Sexually active young people were more likely than their non-sexually active counterparts to speak English at home, to smoke regularly, drink alcohol, binge drink and use marijuana or other illicit substances. They also had greater knowledge about chlamydia infection and testing. Sexually active young people reported that they would be willing to have a chlamydia test if their doctor recommended it, or to protect their own or their sexual partner/s’ health. Barriers to testing included embarrassment, concerns about confidentiality, cost, transport and not having a Medicare card. Most preferred to see a general practitioner (GP) for chlamydia testing.

Compared to Census data from 2006 (the closest year to our study) [15], young people living in major cities, born in Australia and speaking English at home were over-represented but differences were minor. Ninety-one per cent of our sample were engaged in education or paid employment, which is higher than comparable national data from 2007 which found 83.6% of 15 – 24 year old Australians fully engaged in education or training and/or work [16]. We found that being Aboriginal and/or Torres Strait Islander was a predictor of sexual debut < 16 years. This is entirely consistent with sexual experience reported by the first systematic survey of child health among Aboriginal children and young people in Western Australia in 2005 [17]. Reasons for earlier sexual activity are thought to reflect earlier social maturity and the cultural acceptability of earlier child bearing [17]. Rates of substance use in this sample are difficult to compare with either the 2007 national drug strategy household survey [18] or the 2008 national survey of secondary students and sexual health [14] because of different age groupings, definitions, and wording of questions, however there were no stark differences. In the national drug strategy household survey, 32.4% of 14 – 29 year olds smoked daily or weekly, compared to 24.2% of our sample of 16 – 25 year olds. Patterns of alcohol use were very similar between our sample for those aged 16 and 17 and the 2008 National Secondary Schools and Sexual Health survey [14]. Rates of marijuana and other illicit substance use were reasonably congruent between our sample and the national household survey [18]. The correlation between being sexually active and substance use and the clustering of health risk behaviours are consistent with international studies; [19] this is one of very few Australian studies [20] to report explicitly on this.

The high proportion who had had a recent positive chlamydia test is not surprising. We would expect those who had reason to be concerned to be searching for relevant information and come across our website. Therefore it was pleasing that the majority had *not* had a test and that, although unanticipated, young people who had never had sex also chose to complete the baseline questionnaire. Knowledge about chlamydia was considerably higher for comparable questions than in the national survey of secondary students in 2008 but this may reflect the older age of study participants as well as the bias towards young people interested in chlamydia.

Most young people indicated that they would have a chlamydia test to protect their, or their partner/s’ health. This, combined with the fact that one-third of the sample did not know how to get a chlamydia test, while a smaller proportion expressed concerns about confidentiality suggests that education about access to testing is also warranted.

These findings highlight the important role of GPs in chlamydia control. Over 60% of sexually active young people in this study preferred to see a GP for chlamydia testing and almost all (males as well as females) would be willing to have a test if their doctor recommended it. National general practice surveillance data has shown that the “opportunity to test” for chlamydia is the strongest predictor for being tested [21]. Yet over the same study period as ours (2007 – 2008), Medicare data showed that only 12.5% of sexually active young women and 3.7% of sexually active young men were tested for chlamydia [22]. This lower testing rate among young men is consistent with sex differences found in the United Kingdom, one of few countries with a national screening program [23] and one of few that recommends, as in Australia, that asymptomatic, sexually active young men as well as young women are routinely screened. As with our study, this research also found that young men would find screening in general practice acceptable.

Australian GPs identify lack of time and knowledge as barriers to testing, but they also report concern about ‘patient embarrassment’ as a factor [24,25]. Recent evidence suggests that this concern is misplaced and that most patients are willing to discuss STIs with their GPs [26].

While forty percent of our sample also nominated embarrassment as a barrier, the majority stated that they would be comfortable visiting their doctor or nurse for a chlamydia test. Our finding that less than 50% of young people did not want a sexual history taken contrasts with earlier research among young Australian women which found that having a sexual history taken was a dominant concern [27]. These apparent contradictions might reflect differences in our sample’s experience with chlamydia testing compared with those of the general population. But they highlight nuances associated with, and need for sensitivity when discussing, sexuality. The onus should be on doctors or nurses to raise the issue, explore sexual histories sensitively, explain confidentiality, normalise testing as part of routine health care and recommend testing when appropriate. Promoting messages about taking care of one’s health and one’s partner/s’ health might also be effective. We also acknowledge that our data exploring attitudes to testing are now a few years old. Awareness of chlamydia might have increased significantly due to national media campaigns. Young people’s use of technology and social media might have changed considerably since we conducted this study. Whilst the impact of these changes might inform new health promotion strategies, we believe that our findings clearly support that having health professionals directly offer testing opportunistically is likely to be effective at increasing testing rates.

The main limitation of this study is the unique sampling method which brings into question the study’s external validity. Although we believe this was an innovative way to deliver a behavioural intervention, it is a self-selected sample likely to be more interested in chlamydia. While the sample is not generalisable in the same way as a random sample would be, we can gain a sense of how well basic demographic groups are represented (gender, indigenous status, country of birth, region of residence). Website traffic can only infer possible reach of a website rather than provide a denominator for measuring participation rates, which is another challenge in online research. Another limitation of this study is the reliance on self-report: this may have under-estimated exposure to STIs and/or over-estimated screening. However the anonymity of the internet might have facilitated disclosure of personal information.

In summary, Australian young people begin partnered sexual activity in their mid-teen years, and many are at risk of chlamydia and other STIs. The availability of affordable testing and treatment for chlamydia, as well as young people’s willingness to consult with doctors and nurses in general practice and other primary care settings and take up testing, should make it a routine part of all consultations with young people.

## Conclusions

Where one health risk behaviour is identified, it is important to seek information about others. Chlamydia testing can be facilitated by doctors and nurses recommending it. Primary care providers have a useful role in chlamydia control.

## Competing interests

None of the authors have any financial or non-financial competing interests in the publication of this manuscript.

## Authors’ contributions

MK wrote the original grant application, designed and managed the research project, convened and facilitated the Youth Consultant team meetings, promoted the website via media interviews and advertising, assisted with delivering the intervention, conducted most of the data analysis and wrote and revised drafts of the manuscripts. AR was the main clinician involved in delivering the intervention and assisted with data collection. SRS assisted with the grant application, research design, interpretation of data analysis and review of the manuscript. AM assisted with the grant application, research design, interpretation of data analysis and review of the manuscript. MW assisted with the grant application, assisted with facilitation of Youth Consultant team meetings, provided expert advice on e-technology and the use of websites for health promotion and contributed to the interpretation of findings and manuscript review. JP performed a substantial amount of data analysis and assisted with interpretation of statistical findings. TU assisted with research design, interpretation of findings and manuscript review. All authors read and approved the final manuscript.

## Pre-publication history

The pre-publication history for this paper can be accessed here:

http://www.biomedcentral.com/1471-2458/14/12/prepub
